# Lactoferrin-Hydroxyapatite Containing Spongy-Like Hydrogels for Bone Tissue Engineering

**DOI:** 10.3390/ma12132074

**Published:** 2019-06-27

**Authors:** Ana R. Bastos, Lucília P. da Silva, F. Raquel Maia, Sandra Pina, Tânia Rodrigues, Filipa Sousa, Joaquim M. Oliveira, Jillian Cornish, Vitor M. Correlo, Rui L. Reis

**Affiliations:** 1 3B’s Research Group, I3Bs—Research Institute on Biomaterials, Biodegradables and Biomimetics, University of Minho, Headquarters of the European Institute of Excellence on Tissue Engineering and Regenerative Medicine, AvePark, Parque de Ciência e Tecnologia, Zona Industrial da Gandra, 4805-017 Barco, Guimarães, Portugal; 2 ICVS/3B’s—PT Government Associated Laboratory, 4710-057 Braga, Portugal; 3 The Discoveries Centre for Regenerative and Precision Medicine, Headquarters at University of Minho, Avepark, 4805-017 Barco, Guimarães, Portugal; 4 Department of Medicine, University of Auckland, Auckland 1023, New Zealand

**Keywords:** gellan gum, hydroxyapatite, lactoferrin, bone biomaterials

## Abstract

The development of bioactive and cell-responsive materials has fastened the field of bone tissue engineering. Gellan gum (GG) spongy-like hydrogels present high attractive properties for the tissue engineering field, especially due to their wide microarchitecture and tunable mechanical properties, as well as their ability to entrap the responsive cells. Lactoferrin (Lf) and Hydroxyapatite (HAp) are bioactive factors that are known to potentiate faster bone regeneration. Thus, we developed an advanced three-dimensional (3D) biomaterial by integrating these bioactive factors within GG spongy-like hydrogels. Lf-HAp spongy-like hydrogels were characterized in terms of microstructure, water uptake, degradation, and concomitant release of Lf along the time. Human adipose-derived stem cells (hASCs) were seeded and the capacity of these materials to support hASCs in culture for 21 days was assessed. Lf addition within GG spongy-like hydrogels did not change the main features of GG spongy-like hydrogels in terms of porosity, pore size, degradation, and water uptake commitment. Nevertheless, HAp addition promoted an increase of the pore wall thickness (from ~13 to 28 µm) and a decrease on porosity (from ~87% to 64%) and mean pore size (from ~12 to 20 µm), as well as on the degradability and water retention capabilities. A sustained release of Lf was observed for all the formulations up to 30 days. Cell viability assays showed that hASCs were viable during the culture period regarding cell-laden spongy-like hydrogels. Altogether, we demonstrate that GG spongy-like hydrogels containing HAp and Lf in high concentrations gathered favorable 3D bone-like microenvironment with an increased hASCs viability with the presented results.

## 1. Introduction

Over recent years, tissue engineering (TE) has been pushing forward bone tissue regeneration field, especially in what concerns biomaterials’ improvement. Further, the main struggle of bone tissue engineering is to reach a synergistic effect through the combination of biomaterials and cells [1]. Researchers have been demonstrating the importance of biochemical and microstructure cues in the success of designed biomaterials [2]. An optimal biomaterial should not only provide an adequate structural support for bone tissue engineering, but also promote tissue re-growth [3]. The essential prerequisites include: (i) a three dimensional (3D) porous structure, presenting (ii) optimized surface properties, which will potentiate cell attachment, migration, proliferation, and the retention of normal cell functions; (iii) sustained biodegradability; (iv) biomechanical properties that mimic the native tissue; (v) reproducibility; and, (vi) bioactivity, to replace large cortical bone defects and enable load transmission [4,5,6,7]. 

Lf is an iron-binding glycoprotein from the transferrin family [8]. Lf is well known by its antimicrobial, antibacterial, antiviral, antiparasitic anti-neoplastic, anti-inflammatory, and immunomodulatory activities on the immune system, which is a plus for every approach [9,10,11]. Nowadays, despite the molecular mechanism and action of this protein not being completely understood, Lf alone or combined with others systems has been explored for bone tissue engineering [3,12]. Lf alone has been showed to promote the proliferation of rat osteoblasts in a dose and time dependent manner [13]. The integration of recombinant human Lf and MC3T3-E1 osteoblast-like cells in an injectable hydrogel has been shown to enable MC3T3-E1 cells viability, proliferation, and differentiation supporting proteins phosphorylation/dephosphorylation [14]. In vivo systemic administration of Lf alone [15,16] or in a gelatin hydrogel [17] has also been shown to enhance the new bone formation on a bone defect site.

Hydroxyapatite (HAp) (Ca_10_(PO_4_)_6_(OH)_2_) presents a huge similarity to the inorganic component of the bone matrix and it has been widely used in bone tissue engineering as bone graft materials, coatings for implants, and bone fillers due to its ability to directly bond to the deficient apatite layer of the bone through the carbonated calcium [18,19,20,21]. HAp also presents osteoconductive and osteoinductive properties, as it facilitates the migration, adhesion, proliferation, and differentiation of progenitor cells, and the cell-mediated release of growth factors that stimulate bone formation in vivo [22]. We have previously developed Gellan Gum (GG) spongy-like hydrogels that contain HAp for bone tissue engineering [23,24]. GG spongy-like hydrogels show optimal conditions for Tissue Engineering and Regenerative Medicine (TERM) due to their porous microstructure arrangement, mechanical stability, as evidenced by their high flexibility and resilience to deformation, and high water content that altogether potentiate cell adhesion and spreading [25,26,27]. The addition of HAp (10 and 20% w/v) and CaCl_2_, as a crosslinker, to the GG spongy-like hydrogels, improved the bioactivity of the materials, as evidenced by the formation of uniformly distributed apatite-like crystal phases on materials surface in a osteogenic medium, being similar in terms of composition and structure to bone-apatite [24]. In osteogenic culture conditions, HAp-containing spongy-like hydrogels prompted hASCs adhesion and spreading [24], as well as the differentiation of bone marrow cells that were isolated from mice long bones towards pre-osteoclasts [23] in the presence of vitamin D3. 

The combination of Lf and HAp has been scarcely explored for bone tissue engineering. From the few existing in vitro studies, Lf-Hap have shown promising results, as evidenced by enhanced osteoblasts proliferation [3,28] and rADSCs differentiation towards osteoblasts [29]. Hence, in this work, we combined the unique properties of Lf and HAp in spongy-like hydrogels to reinforce the bone-like microenvironment. Different Lf-HAp spongy-like hydrogels formulations were developed and characterized, envisaging a material with appropriate physical-chemical properties and Lf temporal release for future bone tissue engineering approaches. Human adipose-derived stem cells (hASCs) were seeded within the Lf-Hap spongy-like hydrogels due to their promising features, such as their regenerative potential, self-renewal properties, capacity to differentiate into the osteogenic lineage, as well as their high and easy availability from the subcutaneous liposuction from adipose/fat tissue [30,31]. The ability of these biomaterials to behave as a platform to support hASCs cell adhesion and growth was then studied.

## 2. Materials and Methods

### 2.1. Preparation of Hydrogels

Gelzan CM (Sigma, St. Louis, MO, USA) was dissolved in distilled water at 90 °C for 30 min. with stirring. Lactoferrin (Lf, Bovine origin, New Zealand) was dissolved in distilled water. Subsequently, to the solution of Gellan Gum (GG), Lactoferrin (Lf), and Hydroxyapatite (HAp, Plasma Biotal, Buxton, UK) were added at 50 °C, as described in Table 1. After complete homogenization, the crosslinking solution CaCl_2_ (Sigma, USA) was added and it was allowed to stabilize at room temperature for 30 min. Posteriorly, the hydrogels were cut in discs of 5 mm of diameter and 2 mm of thickness and replenished with phosphate-buffered saline solution (PBS, Sigma, USA) for 30 h to enable complete crosslinking, frozen at −80 °C for 18–20 h, and then freeze-dried (CryoDos -80, Telstar, Terrassa, Barcelona, Spain) for at least three days. Materials were sterilized by ethylene oxide.

### 2.2. Physico-Chemical Characterization of Spongy-Like Hydrogels’

#### 2.2.1. Fourier Transformed Infrared Spectroscopy (FTIR)

Fourier Transformed Infrared (FTIR) analysis was performed while using attenuated total reflectance (ATR) (IRPrestige-21, Shimadzu Corporation, Kyoto, Japan) in transmittance mode and in the region of 550–4000 cm^−1^ for spectroscopic study. 

#### 2.2.2. X-ray Diffraction (XRD) Analysis

The qualitative analyses of crystalline phases that were presented on the GG, GG/Lf, GG/HAp, and GG/Lf/HAp spongy-like hydrogels were obtained by XRD while using a conventional Bragg–Brentano diffractometer (Bruker D8 Advance DaVinci, Rheinstetten, Germany) that was equipped with CuKα radiation, produced at 40 kV and 40 mA. The data sets were collected in the 2θ range of 5–70° with a step size of 0.04° and 1 s for each step.

#### 2.2.3. Scanning Electron Microscopy 

Scanning electron microscopy (SEM, JSM-6010 LV, JEOL, Tokyo, Japan) and micro computed tomography (micro-CT) were used to assess the microstructure of the dried polymeric networks of the different formulations. For SEM analysis, the samples were immersed in liquid nitrogen to cut the sample into cross-sections, and the internal surface of the dried polymeric networks was observed. 

#### 2.2.4. Micro Computed Tomography

The microstructure of dried polymeric networks was analyzed while using a high resolution X-Ray microtomography SkyScan 1272 System (SkyScan, Kontich, Belgium). The pixel size used was 2.5 μm, the exposure time was 1s, and the source conditions used were 50 kV of energy and 200 μA of current. A binary picture was created using 150 slides while using a thresholding between 20 and 255 in a grey scale. The morphometric analysis, which includes porosity, pore size, interconnectivity, and wall thickness, were assessed by CT-analyzer program (CTAn, v1.17.0.0., SkyScan, Belgium). 

#### 2.2.5. Degradation Studies: Mass Loss

Lf-HAp containing spongy-like hydrogels were immersed in PBS at 37 °C for 30 days, with stirring. The initial weight (w_i_) of the samples was recorded and the samples were then immersed in PBS and weighed again (final weight, w_f_). The percentage of mass loss along the time was calculated according to the equation:

$$Mass\;loss\left(\%\right)=\frac{\left(W_{f}-W_{i}\right)}{W_{i}}\times 100$$

Every three days, the PBS solution was replaced and the supernatant was collected and then kept at −80 °C for further analysis of the amount of Lf released.

#### 2.2.6. Water Uptake

Lf-HAp containing spongy-like hydrogels were weighed in the dried state (w_d_) and were then immersed in PBS at 37 °C during three days. Along this period of time, at different time points (30 min., 1, 2, 4, 6, 24, 48, and 72 h), the samples were weighed in the wet state (w_w_). The percentage of water uptake along the time was calculated according to the equation:

$$Water\;Uptake\left(\%\right)=\frac{\left(W_{w}-W_{d}\right)}{W_{d}}\times 100$$

#### 2.2.7. Quantification of Lf Release: BCA Assay 

A micro-Bicinchoninic Acid Assay (micro-BCA) was performed according to manufacturer instructions In order to evaluate the amount of Lf released from spongy-like hydrogels. Briefly, 75 µL of bicinchoninic acid was placed in each well of a 96 well-plate and 75 µL of the supernatant, previously collected during mass loss assessment, was added to each one. These well-plates were incubated for 2 h at 37 °C. After this time, the absorbance at 562 nm was measured while using a Microplate Reader (SYNERGY HT, BIO-TEK, USA). The supernatants from GG and GG with HAp spongy-like hydrogels were used as Blank. The corrected absorbance readings were converted into protein concentrations while using a calibration curve of Lf. The theoretical value according to the final volume and concentration of Lf into GG spongy-like hydrogels was calculated. GG/Low Lf containing spongy-like hydrogels, according to the theoretical calculations, can release a maximum of 20 µg, while G/High Lf can release a maximum of 60 µg.

### 2.3. In Vitro Studies

#### 2.3.1. Cell Isolation and Culture

Human adipose-derived stem cells (hASCs) were isolated from the lipoaspirate samples following a protocol previously established with the Department of Plastic Surgery of Hospital da Prelada (Porto, Portugal). All subjects gave their informed consent for inclusion before they participated in the study. The study was conducted in accordance with the Declaration of Helsinki, and the protocol was approved by the Ethics Committee of Hospital da Prelada (P.I. Nº 005/2019) and 3B’s Research Group. The isolated cells were used for subsequent studies. The cells were routinely cultured in α-MEM medium (Alfagene, Carcavelos, Portugal) that was supplemented with 10% fetal bovine serum (FBS, Australia origin, Alfagene, Portugal), 1% antibiotic/antimycotic (Alfagene, Portugal), and maintained at 37 °C under a humidified atmosphere of 5% v/v CO_2_ in air. The medium was changed twice a week and cells, at maximum passage 4, were seeded into spongy-like hydrogels, as described in the next section. 

#### 2.3.2. Cell Entrapment

A cell suspension (40 × 10^3^, 20 µL) was dispensed dropwise on the top of the polymeric networks, which were previously hydrated for 15 min. with culture medium. Posteriorly, these constructs were incubated at 37 °C during 3 h, with 5% CO_2_ to allow for maximum cell entrapment within the structures. Fresh medium (500 µL) was added after this period of time.

#### 2.3.3. Cell Viability

Cell viability was assessed at 21 days while using 20% (v/v) of Alamar Blue reagent (ALAMAR BLUE^®^, AbD, Kidlington, Oxford, UK) in α-MEM culture medium, followed by 3 h of incubation at 37 °C with 5% CO_2_ and protected from light. Afterwards, 100 µL of supernatant was transferred from each well in triplicate to a new 96-well cell culture plate. Fluorescence intensity was read at 530/20 nm (excitation) and 590/35 nm (emission) using a microplate reader (Synergy HT, Bio-Tek, USA). Alamar Blue in medium was used as a blank. DNA normalized the corrected absorbance readings at day 21. Three specimens of each formulation were used to assess cell viability along the time and three independent experiments were performed. 

#### 2.3.4. DNA Content

DNA content was assessed after 21 days of culture and it was used to normalize the cell viability values of the same time point. Constructs were washed with PBS and transferred to 1.5 mL tubes and heated at 70 °C for 30 min. Next, 1 mL of ultra-pure water was added into each tube that were placed at 37 °C for 1 h, and then frozen at −80 °C until analysis. Before DNA quantification, the samples were placed in an ultrasound bath for 1 h at 37 °C. Finally, Quant-IT PicoGreen dsDNA Assay Kit 2000 assays (Alfagene^®^, Portugal) was used according to the manufacturer’s instructions. The fluorescence intensity was read at 485/20 nm (excitation) and 530/20 nm (emission) while using a microplate reader (Synergy HT, Bio-Tek, Winooski, VT, USA) and the readings were converted while using a standard curve that was produced with standard dsDNA solutions at different concentrations. 

#### 2.3.5. Cytoskeleton Morphology (Phalloidin/DAPI)

After three and 21 days of culture, the constructs were washed with PBS during 5 min., fixed with 10% formalin for one hour, and then washed again. Next, Phalloidin-TRITC (Sigma, USA) (1:80) was added and placed at room temperature during 1 h, protected from light. The constructs were counterstained with 4,6-Diamidino-2-phenylindole, Dilactate (DAPI, Sigma, USA) (1:5000) for 5 min. in the dark. Finally, they were visualized by Confocal Laser Scanning Microscope (TCS SP8, Leica, Mannheim, Germany).

### 2.4. Statistics

Statistical analysis was performed while using the GraphPad Prism 5.0 software. The data were analyzed while using the Shapiro–Wilk normality test. The results that did not present a normal distribution were analyzed using the Kruskal–Wallis test with Dunn’s multiple comparison post-test for statistical analysis. The results that presented a normal distribution were analyzed while using a One-way ANOVA and Tukey’s multiple comparisons T-test. The significance level between the groups were set for * *p* < 0.05, ** *p* < 0.01, and *** *p* < 0.001. The data were presented as mean ± standard deviation (SD). Data in each figure are representative experiment of three experiments (n = 3).

## 3. Results

### 3.1. FTIR and XRD Analysis 

**Figure 1:**
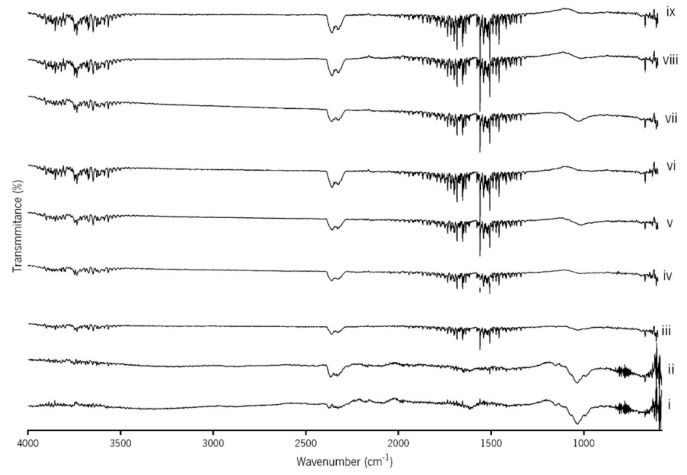
Fourier Transformed Infrared (FTIR) spectra of GG/Lf/HAp spongy-like hydrogels: (i) GG, (ii) GG/Low Lf, (iii) GG/High Lf, (iv) GG/Low HAp, (v) GG/High HAp, (vi) GG/Low Lf/Low HAp, (vii) GG/High Lf/Low HAp, (viii) GG/Low Lf/High HAp, and (ix) GG/High Lf/High HAp.

The ATR-FTIR spectra of the spongy-like hydrogels that are presented in Figure 1 show the stretching vibrations of C–O–C bonds and the bending mode of mehtyl vC–H, respectively, at 1022 and 1593 cm^−1^ [32]. Regarding the Lf addition to the GG formulations, a signal of tyrosine was registered at 1138 cm^−1^ [33]. Additionally, an absorption peak appears at 1010 cm^−1^, which can be assigned to N≡C or C=C stretch and C–H deformation vibrations of tryptophan. The presence of HAp was ascertained by the detection of the peak around 600 cm^−1^, in the region of the v4 bending mode of PO_4_^3−^ and the peak around 1000 cm^−1^ that corresponded to the region of the v1 stretching mode of PO_4_^3−^ [24]. All of the GG/Lf/HAp spongy-like hydrogels show the broad band between 3500 and 3700 cm^−1^ that is attributed to the hydroxyl groups (vOH) stretching vibration due to the medium hydrogen bond of intramolecular and intermolecular type. 

**Figure 2:**
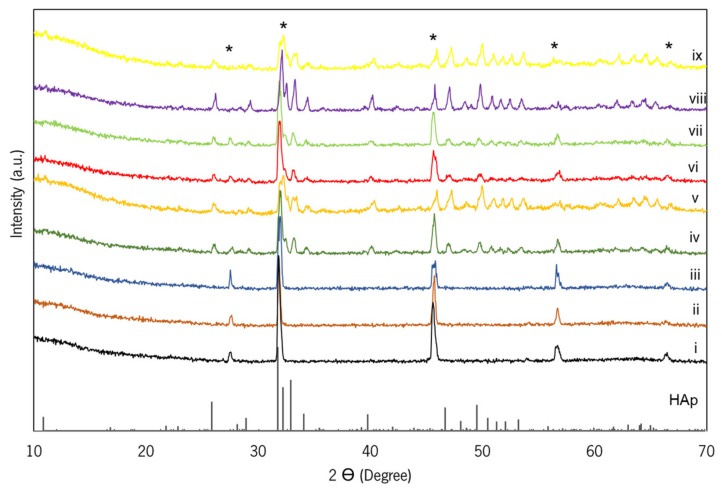
X-ray Diffraction (XRD) patterns of the GG/Lf/HAp spongy-like hydrogels: (i) GG, (ii) GG/Low Lf, (iii) GG/High Lf, (iv) GG/Low HAp, (v) GG/High HAp, (vi) GG/Low Lf/Low HAp, (vii) GG/High Lf/Low HAp, (viii) GG/Low Lf/High HAp and (ix) GG/High Lf/High HAp. HAp (ICDD PDF 01-074-0565), and * GG.

Figure 2 displays the XRD patterns of the spongy-like hydrogels. It can be observed that all of the compositions show the intensity peaks corresponding to the GG crystalline structure with the main peak being located at 32° [32]. The presence of a higher content of Lf led to a slight crystallinity decrease without additional peaks being observed (Figure 2iii). The crystallographic phases identification of the hydrogels containing HAP was accomplished by comparing the XRD patterns with the standard ICDD PDF 01-074-0565 of HAp (Figure 2iv–ix). As expected, with increasing the HAp content in the hydrogels, the crystallinity became more evident, as shown in Figure 2v,viii,ix.

### 3.2. SEM and Micro-CT Analysis

**Figure 3:**
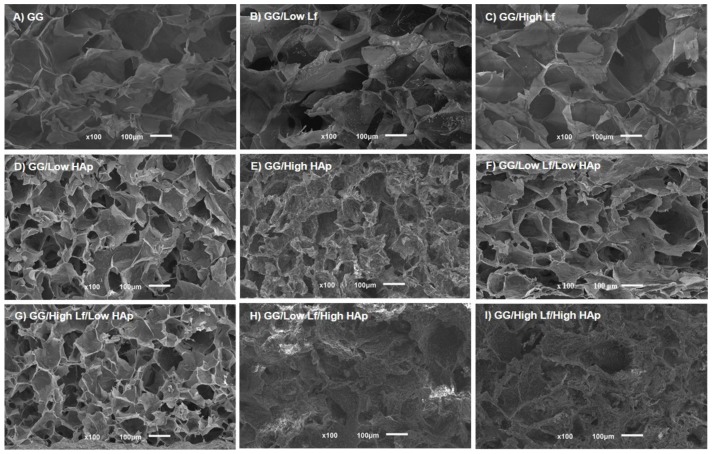
SEM representative images of GG dried polymeric networks (DPN) with or without Lactoferrin (Lf) and/or Hydroxyapatite (HAp): (**A**) GG, (**B**) GG/Low Lf, (**C**) GG/High Lf, (**D**) GG/Low HAp, (**E**) GG/High HAp, (**F**) GG/Low Lf/Low HAp, (**G**) GG/High Lf/Low HAp, (**H**) GG/Low Lf/High HAp, and (**I**) GG/High Lf/High HAp.

Figure 3 shows the representative images of the cross section surface of the different GG/Lf/HAp dried polymeric networks (DPN) that were obtained by SEM. GG DPN containing just Lf (Low or High) showed a porous structure with smooth surfaces, which indicated that the presence of Lf had no effect in the microstructure when compared with the control (GG) (Figure 3A–C). Regarding GG DPN containing HAp (Figure 3D,E), smaller pores and rough surfaces were observed. This effect was more evident in the formulations containing higher HAp concentrations (1% in relation to 10%). The microstructure of GG DPN containing Lf/HAp was similar to the respective GG/HAp DPN (Figure 3F,G in relation to Figure 3D; Figure 3H,I in relation to Figure 3E). These results indicate that no evident effect was observed by adding Lf, while the addition of HAp affected the microstructure.

**Figure 4:**
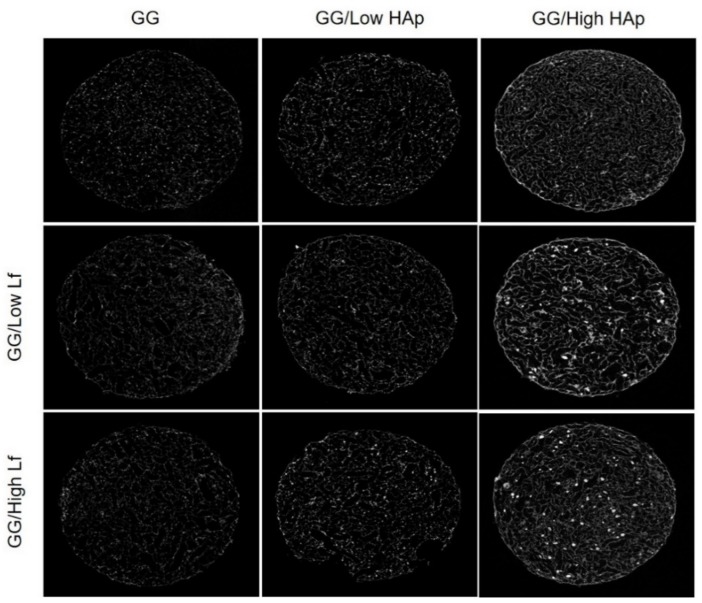
Two-dimensional (2D) images of the dried polymer networks of GG/Lf/HAp spongy-like hydrogels obtained by Micro-CT.

Porosity, pore wall thickness, and average pore size were quantified by micro-CT analysis (Table 2). The obtained results revealed that the addition of HAp has an inverse effect on the porosity of the DPN: the porosity tended to decrease with the increase of HAp concentration from ~87% to 64%, respectively. Despite this tendency, no other statistically significant differences on the average porosity were observed. Nevertheless, the same trend was verified on the mean pore size for all of the conditions: a decrease of pore size was observed when HAp is added. For these formulations, the decrease on pore size was followed by an increase of the pore wall thickness, although no significant differences were observed. In accordance to SEM results, the HAp addition had a higher impact in the microstructure of GG polymeric networks than Lf addition. In addition, the 2D microarrangement of the dried polymeric networks can be observed in Figure 4.

### 3.3. Water Uptake Analysis

**Figure 5:**
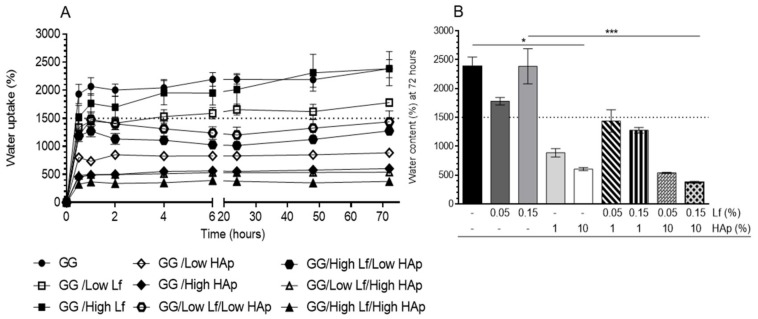
(**A**) Water uptake profile of dried polymeric networks (DPN) along 72 h (**B**) water content after 72 h. Data was presented as mean ± stdev and statistical analysis was performed while using a Kruskal-Wallis test followed by Dunn’s test.

The water uptake of the different GG DNP formulations was followed up to three days of immersion into PBS (Figure 5). All of the formulations have shown a burst of water uptake in the first hours of immersion, followed by an equilibrium phase corresponding to the maximum of water content. GG showed a water content of 2385% ± 357 after three days of immersion (Figure 5B). Similar values were observed for the GG/High Lf formulation. However, GG/Low Lf formulations have shown lower water content (1780% ± 143). All of the formulations containing HAp showed lower water content values (~1500%) when comparing to GG and both GG/Lf formulations. GG spongy-like hydrogels with 1 (GG/Low HAp) and 10% (GG/High HAp) of HAp showed a water content of 886% ± 160 and 603% ± 62, respectively. GG spongy-like hydrogels with 1% of HAp and Lf, independent of the concentration, showed a water content between 1277% ± 109 and 1438% ± 438, while the formulations containing 10% of HAp and Lf, the water content was lower (~300/500%). These results have shown that the addition of HAp at higher concentration (10%) to the GG formulation significantly decreased (*p* < 0.05) their water uptake ability (from 2385% ± 357 to 603% ± 63). Likewise, a significant decrease on water content between GG/High Lf (~2385% ± 683) and GG/High Lf/High HAp (~375% ± 35) (*p* < 0.001) was also observed due to HAp addition.

### 3.4. Degradation Tests and Lf Release Analysis

**Figure 6:**
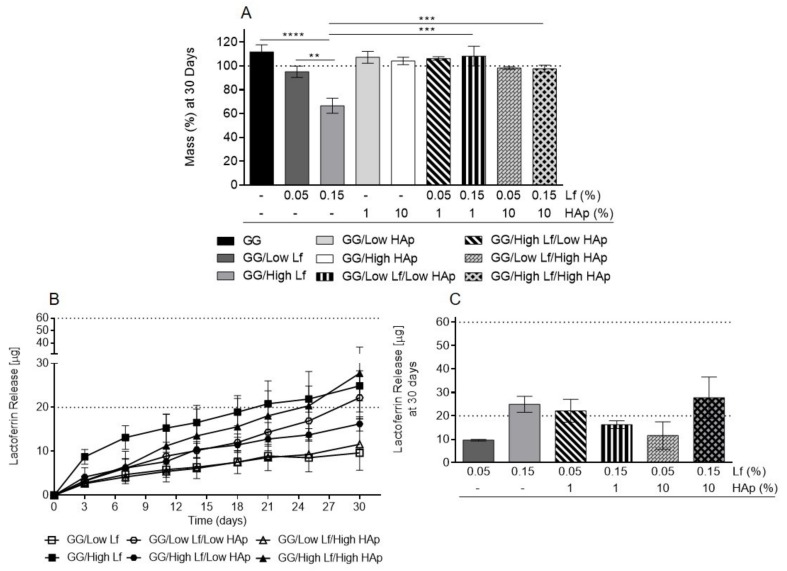
Degradation and Lf release analysis. Final mass of dried polymeric networks after 30 days (**A**). Lf release profile of Lf-HAp containing spongy-like hydrogels along 30 days (**B**) and at day 30 (**C**). The statistical analysis of the final mass was performed using a One-way ANOVA and Tukey’s multiple comparisons T-test, while the Kruskal–Wallis test followed by Dunn’s test was used for Lf release and all the formulations were compared between them.

The degradation of different formulations was followed up to 30 days by quantifying the weight loss (Figure 6A). GG/Low Lf and GG/High Lf spongy-like hydrogels have shown higher mass loss in comparison with the other formulations (Figure 6A). In accordance, the GG/High Lf spongy-like hydrogels showed a significant mass loss when comparing to GG (*p* < 0.05), GG/Low Lf (*p* < 0.01), and Lf/HAp (*p* < 0.001) containing spongy-like hydrogels.

The release of Lf was also analyzed for this period of time (Figure 6B,C). A sustained release of Lf was observed for all the formulations along the 30 days (Figure 6B). As expected, formulations containing 0.15% of Lf presented a higher, but not significant, Lf release when compared with 0.05% of Lf formulations (Figure 6B,C). In addition, the spongy-like hydrogels released half of the existent Lf after 30 days, namely 20 µg for spongy-like hydrogels with 0.05 % Lf and 60 µg for spongy-like hydrogels with 0.15 %, with the exception of GG/Low Lf/Low Hap, which released the total amount of Lf (20 µg).

### 3.5. Cell Viability and Cell Cytoskeleton Analysis

**Figure 7:**
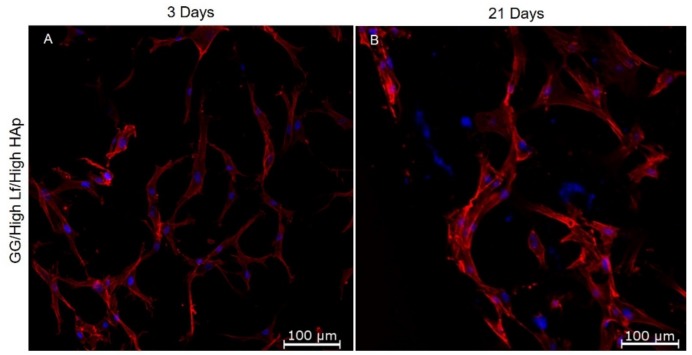
Representative images of cytoskeleton morphology (Phalloidin-TRITC, red) and nuclei (DAPI,blue) of hASCs within GG/High Lf/High HAp spongy-like hydrogels after (**A**) three and (**B**) 21 days of culture.

hASCs’ cytoskeleton within spongy-like hydrogels was analyzed by Phalloidin staining (Figure 7). Independent of the spongy-like hydrogel formulation, cells attached and showed a spread morphology within the polymeric structure from three days of culture onward 21 days. Figure 4 shows cell cytoskeleton organization within GG/High Lf/High HAp, which is representative of the other spongy-like hydrogel formulations. 

**Figure 8:**
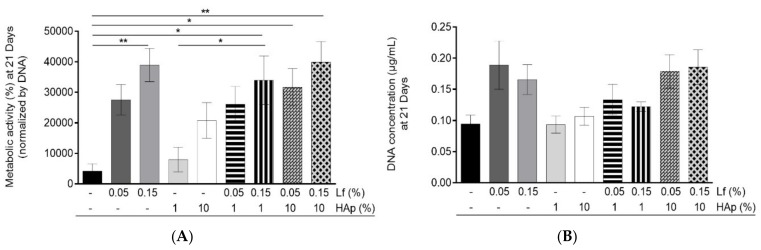
Cell viability assessment. (**A**) Metabolic activity of hASCs’ normalized by Day 1 after 21 days of culture. (**B**) DNA concentration of hASCs’ within spongy-like hydrogels after 21 days. The statistical analysis was performed using a One-way ANOVA and Tukey’s multiple comparisons T-test and results were presented as mean ± standard deviation (SD) and the significance level between groups was set for: * *p* < 0.05, and ** *p* < 0.01.

Cell viability within the spongy-like hydrogels was assessed through Alamar Blue® assay that was normalized by DNA at that time point (21 days) (Figure 8A). It was possible to verify that hASCs remained metabolically active after 21 days of culture. Noteworthy, the formulations containing Lf in combination or not with HAp showed the highest metabolic activity, while GG and GG/Low HAp spongy-like hydrogels showed the lowest metabolic activity. GG/High Lf, GG/High Lf/Low HAp, GG/Low Lf/High HAp, and GG/High Lf/High HAp showed significantly higher metabolic activity when comparing to the GG spongy-like hydrogels. A significant increase in metabolic activity was also verified between GG/Low HAp and GG/High Lf/Low HAp. Regarding the DNA concentration depicted in Figure 5B, it was possible to observe that, after 21 days, the cells were present in all the spongy-like hydrogels tested. Moreover, the results showed that spongy-like hydrogels with Lf combined or not with HAp have higher amounts of DNA, while GG spongy-like hydrogels have the lowest. However, statistical differences were not observed.

## 4. Discussion

New biomaterials, cells, and growth factors are being combined to reach a synergistic effect and produce functional tissue engineered bone substitutes. With this in mind, we developed a batch of novel bioactive GG spongy-like hydrogels that contain different concentrations of Lf and/or HAp, and tested its capacity to support hASCs culture, envisioning its use as improved scaffolds for bone tissue engineering. In fact, hASCs can differentiate along the osteogenic lineage when submitted to specific growth factors, such as Lf [15,16,34].

The biomaterials microarchitecture, including pore size and porosity, are crucial parameters to match the native tissue characteristics and the required integrity [35]. This work showed that the presence of HAp significantly reduced the porosity and pore size. The HAp functioned as a nucleation agent for ice crystals during the freezing step of spongy-like hydrogels preparation, leading to the formation of pores in a higher amount, but with lower sizes [25,27]. Nevertheless, the results that were obtained regarding porosity and pore size, for all of the formulations, are in agreement with the literature that indicate that porosity between 35 and 75% and the pore size in the range of 50 to 400 µm are appropriated to be used in bone tissue engineering applications [36]. In our study, all of the developed spongy-like hydrogels presented pore sizes that were between ~40 to 80 µm, which are within the range that was described to allow for the ingrowth of capillaries and facilitate the exchange of nutrients and discharge of metabolites [1]. In fact, we could verify that hASCs that were cultured within all spongy-like hydrogels were metabolically active. Furthermore, the cells cultured within spongy-like hydrogels with Lf and/or HAp were more metabolic active than cells that were cultured within GG spongy-like hydrogels. This was an expected result, since Gellan Gum hydrogels do not present specific attachment sites for anchorage-dependent cells. When considering the pore size, it was also observed that the addition of HAp lead to a decrease in the porosity while thicker and rough pore walls were promoted. The commitment of this combined effect together with the intrinsic properties of the GG spongy-like hydrogels can overcome the problems that are associated with stress shielding [37]. It is well known that the thicker pore walls and roughness might be crucial for cells biological response since it directly influences cell migration and proliferation [38]. Moreover, it promotes structural support and adhesion sites, facilitates cell movement, regulates cell behavior, and sustains cell-to-cell recognition. 

On the other hand, the microarchitecture of spongy-like hydrogels was not altered with the addition of Lf. In fact, this small protein was predicted to adsorb to the GG polymeric networks and it did not act as a nucleation agent for ice crystals in the freezing stage. GG spongy-like hydrogels Lf containing have shown higher porosity and water retention capacity, when compared with HAp containing spongy-like hydrogels. Similarly, in other studies, it has been verified that, when HAp is incorporated into a matrix, a lower water content is adsorbed/expected [23,39,40]. The achievement of the maximum water content occurs earlier due to the existence of lower microvoids between the interface of the GG matrix and the HAp crystals. Since HAp has very low water absorption, when it is added to the GG matrix, its natural/intrinsic hydrophilicity decreases. Overall, the microstructure of the developed spongy-like hydrogels was proper for the diffusion of oxygen and nutrients, since the cells showed their normal phenotype after 21 days of culture. Furthermore, it was also observed that the pore size did not negatively affect cell spreading and morphology, as demonstrated by cytoskeleton staining.

When considering the bioactivity potential of HAp and/or Lf, the release of these bioactive components to the surrounding environment is of high importance in an in vivo scenario. Hence, the release of these bioactive factors was studied in vitro along 31 days. The serum level of Lf on the human body is around 2 to 7 μg/mL, and it has been applied to achieve the desired effect from the contact of Lf with different cell types (e.g. proliferation and/or the inhibition of bone resorption) [17]. Having this in mind, we applied a protein concentration within our biomaterials that, when released, was similar to the protein concentration that is found in human body. Lf containing spongy-like hydrogels showed a significant mass loss, in contrast to the HAp containing spongy-like hydrogels. This can be explained by the higher release of non-crosslinked Lf from the semi-interpenetrating networks, which causes the disintegration of the polymeric networks. Interestingly, when they were combined, the HAp presence masked the Lf presence in all aspects that were previously mentioned. Perhaps it happens due to the high affinity between HAp and Lf, which is mainly provided by the electrostatic interactions between Lf and HAp surface [3,41]. Montesi et al., also reported that the protein quantity is a crucial parameter, since a lower amount of Lf only interacted with the negative surface ionic groups of HAp, while higher amount of Lf probably favored the protein–protein interactions (through hydrogen or hydrophobic/hydrophilic interactions) and formation of multiple protein layers with different molecular orientations [41]. Furthermore, these interactions were sustained by Lf incorporation during hydrogel preparation, which affected the hydrogel chains formation. It was also reported that the swelling ratio was correlated with the quantity of protein released [42], since a faster desorption of the Lf molecules being non-directly bounded on the particles surface is verified, when the scaffolds are immersed in a hydrated medium. However, in spite of non-existent significant differences, the formulations with 10% of HAp and Lf in both concentrations, showed lower swelling ratio and, consequently, a lower release. As expected, when Lf was present in a high amount, it had more protein to release. Montesi et al. demonstrated that, in addition to the HAp biocompatibility, osteoconductivity, and biodegradability, this bioceramic has the ability to bind several biomolecules without affecting their biological function [41]. Overall, some gaps have been identified based on the existing studies. In fact, most of the existing studies are performed while using animal cells/models hindering the correlation of these models with future clinical outcomes. Moreover, the results that were obtained can be conditioned, depending of the type and size of the defect, the degradation process, and the protein release, which resulted in different bone regeneration rates. 

Our study demonstrates the synergetic effect from the combination of HAp with Lf and hASCs and their ability for use in spongy-like hydrogels for bone tissue engineering applications.

## 5. Conclusions

Bone tissue engineering has been explored by combining new biomaterials and cells to reach a synergistic effect. With this in mind, we studied the effect from the combination of bioactive compounds, Lf and HAp, within GG spongy-like hydrogels to be used in the bone tissue regeneration approaches. 

The parameters studied, such as porosity, pore size, pore wall thickness, weight loss, water uptake, and Lf release, were tailored through the addition and/or combination of each compound. Lf containing spongy-like hydrogels showed similar features with GG spongy-like hydrogels. In contrast, differences in terms of all parameters studied were observed in GG/HAp and Lf-HAp containing spongy-like hydrogels. The HAp addition promoted an increase of the wall thickness and a decrease on porosity and pore size as well as, on degradability and water retention capabilities. Related to the Lf release, along the 30 days all formulations released at least half of the existent lactoferrin in both (0.05 and 0.15%) of the formulations containing Lf. Moreover, the capacity of these biomaterials to support hASCs in culture for 21 days was assessed. The microstructure of Lf/HAp-containing spongy-like hydrogels seemed to be proper for the diffusion of oxygen and nutrients, since the cells spread and showed their normal phenotype after 21 days. In conclusion, Lf and HAp in high concentrations assembled the required conditions to conduce these biomaterials to promote bone regeneration. 

## Figures and Tables

**Table 1:** Different formulations of Gellan Gum (GG) spongy-like hydrogels with or without Lactoferrin (Lf), and/or Hydroxyapatite (HAp).

Name	GG % (w/v)	Lf % (w/v)	HAp % (w/v)
**GG**	1.25	-	-
**GG/Low Lf**	1.25	0.05	-
**GG/High Lf**	1.25	0.15	-
**GG/Low HAp**	1.25	-	1.00
**GG/High HAp**	1.25	-	10.00
**GG/Low Lf/ Low HAp**	1.25	0.05	1.00
**GG/High Lf/Low HAp**	1.25	0.15	1.00
**GG/Low Lf/High HAp**	1.25	0.05	10.00
**GG/High Lf/High HAp**	1.25	0.15	10.00

**Table 2:** Micro computed tomography (Micro-CT) analysis of (A) mean porosity, (B) pore size, and (C) wall thickness of GG dried polymeric networks (DPN) with or without Lf and/or HAp. Data was presented as mean ± stdev, the statistical analysis was performed using a Kruskal–Wallis test followed by Dunn’s test, in which all formulations were compared between them.

Name	Porosity (%)	Pore Size (µm)	Wall Thickness (µm)
**GG**	86.5 ± 1.34	73.18 ± 12.92	12,83 ± 0.87
**GG/Low Lf**	86.01 ± 1.81	73.14 ± 5.37	13.21 ± 0.14
**GG/High Lf**	86.98 ± 1.97	68.85 ± 0.93	13.45 ± 1.31
**GG/Low HAp**	78.44 ± 1.37	55.23 ± 5.22	17.43 ± 0.21
**GG/High HAp**	64.45 ± 3.79	42.80 ± 5.73	20.17 ± 0.66
**GG/Low Lf/ Low HAp**	81.30 ± 0.64	63.30 ± 3.66	16.07 ± 0.42
**GG/High Lf/Low HAp**	84.14 ± 3.21	79.06 ± 15.31	16.48 ± 0.62
**GG/Low Lf/High HAp**	67.98 ± 0.55	59.96 ± 3.53	27.95 ± 1.66
**GG/High Lf/High HAp**	71.22 ± 2.96	59.74 ± 2.00	25.66 ± 1.72

## References

[1] Gao C., Peng S., Feng P., Shuai C. (2017). Bone biomaterials and interactions with stem cells. Bone Res..

[2] Black C.R.M., Goriainov V., Gibbs D., Kanczler J., Tare R.S., Oreffo R.O.C. (2015). Bone Tissue Engineering. Curr. Mol. Biol. Rep..

[3] Shi P., Wang Q., Yu C., Fan F., Liu M., Tu M., Lu W., Du M. (2017). Hydroxyapatite nanorod and microsphere functionalized with bioactive lactoferrin as a new biomaterial for enhancement bone regeneration. Colloids Surf. B Biointerfaces.

[4] Henkel J., Woodruff M.A., Epari D.R., Steck R., Glatt V., Dickinson I.C., Choong P.F.M., Schuetz M.A., Hutmacher D.W. (2013). Bone Regeneration Based on Tissue Engineering Conceptions—A 21st Century Perspective. Bone Res..

[5] Kumar S.D. (2015). Study of Development and Applications of Bioactive Materials and Methods In Bone Tissue Engineering. Biomed. Res..

[6] Silva S.S., Fernandes E.M., Silva-Correia J., Pina S.C.A., Vieira S., Oliveira J.M., Reis R.L., Ducheyne P., Healy K., Hutmacher D.W., Grainger D.W., Kirkpatrick C.J. (2017). Natural-Origin Materials for Tissue Engineering and Regenerative Medicine. Comprehensive Biomaterials II.

[7] Matassi F., Nistri L., Paez D.C., Innocenti M. (2011). New biomaterials for bone regeneration. Clin. Cases Miner. Bone Metab..

[8] Lönnerdal B. (1995). Lactoferrin: Molecular Structure and Biological Function. Annu. Rev. Nutr..

[9] Farnaud S., Evans R.W. (2003). Lactoferrin—A multifunctional protein with antimicrobial properties. Mol. Immunol..

[10] Gao R., Watson M., Callon K.E., Tuari D., Dray M., Naot D., Amirapu S., Munro J.T., Cornish J., Musson D.S. (2018). Local application of lactoferrin promotes bone regeneration in a rat critical-sized calvarial defect model as demonstrated by micro-CT and histological analysis. J. Tissue Eng. Regen. Med..

[11] Bruni N., Capucchio M.T., Biasibetti E., Pessione E., Cirrincione S., Giraudo L., Corona A., Dosio F. (2016). Antimicrobial Activity of Lactoferrin-Related Peptides and Applications in Human and Veterinary Medicine. Molecules.

[12] Montesi M., Panseri S., Iafisco M., Adamiano A., Tampieri A. (2015). Coupling Hydroxyapatite Nanocrystals with Lactoferrin as a Promising Strategy to Fine Regulate Bone Homeostasis. PLoS ONE.

[13] Jun W., Bi-Yu Z., Chuang-Yue Z. (2016). Effect of lactoferrin on rat osteoblast proliferation. J. Hainan Med. Univ..

[14] Amini A.A., Nair L.S. (2014). Recombinant human lactoferrin as a biomaterial for bone tissue engineering: Mechanism of antiapoptotic and osteogenic activity. Adv. Healthc. Mater..

[15] Yoshimaki T., Sato S., Tsunori K., Shino H., Iguchi S., Arai Y., Ito K., Ogiso B. (2013). Bone regeneration with systemic administration of lactoferrin in non-critical-sized rat calvarial bone defects. J. Oral Sci..

[16] Li W., Zhu S., Hu J. (2015). Bone Regeneration Is Promoted by Orally Administered Bovine Lactoferrin in a Rabbit Tibial Distraction Osteogenesis Model. Clin. Orthop. Relat. Res..

[17] Takaoka R., Hikasa Y., Hayashi K., Tabata Y. (2011). Bone Regeneration by Lactoferrin Released from a Gelatin Hydrogel. J. Biomater. Sci. Polym. Ed..

[18] Prakasam M., Locs J., Salma-Ancane K., Loca D., Largeteau A., Berzina-Cimdina L. (2015). Fabrication, Properties and Applications of Dense Hydroxyapatite: A Review. J. Funct. Biomater..

[19] Tayyebi S., Mirjalili F., Samadi H., Nemati A. (2015). A Review of Synthesis and Properties of Hydroxyapatite/Alumina Nano Composite Powder. Chem. J..

[20] Haider A., Haider S., Han S.S., Kang I.-K. (2017). Recent advances in the synthesis, functionalization and biomedical applications of hydroxyapatite: A review. RSC Adv..

[21] Kattimani V.S., Kondaka S., Lingamaneni K.P. (2016). Hydroxyapatite—Past, Present, and Future in Bone Regeneration. Bone Tissue Regen. Insights.

[22] Wang W., Yeung K.W. (2017). Bone grafts and biomaterials substitutes for bone defect repair: A review. Bioact. Mater..

[23] Maia F.R., Musson D.S., Naot D., Da Silva L.P., Bastos A.R., Costa J.B., Oliveira J.M., Correlo V.M., Reis R.L., Cornish J. (2018). Differentiation of osteoclast precursors on gellan gum-based spongy-like hydrogels for bone tissue engineering. Biomed. Mater..

[24] Manda M.G., da Silva L.P., Cerqueira M.T., Pereira D.R., Oliveira M.B., Mano J.F., Marques A.P., Oliveira J.M., Correlo V.M., Reis R.L. (2018). Gellan gum-hydroxyapatite composite spongy-like hydrogels for bone tissue engineering. J. Biomed. Mater. Res. Part A.

[25] Gantar A., Da Silva L.P., Oliveira J.M., Marques A.P., Correlo V.M., Novak S., Reis R.L. (2014). Nanoparticulate bioactive-glass-reinforced gellan-gum hydrogels for bone-tissue engineering. Mater. Sci. Eng. C.

[26] Cerqueira M.T., da Silva L.P., Correlo V.M., Reis R.L., Marques A.P. (2015). Epidermis recreation in spongy-like hydrogels: New opportunities to explore epidermis-like analogues. Mater. Today.

[27] Da Silva L.P., Cerqueira M.T., Sousa R.A., Reis R.L., Correlo V.M., Marques A.P. (2014). Engineering cell-adhesive gellan gum spongy-like hydrogels for regenerative medicine purposes. Acta Biomater..

[28] Ohsugi H., Habuto Y., Honda M., Aizawa M., Kanzawa N. (2013). Evaluation of the Anti-Bacterial Activity of a Novel Chelate-Setting Apatite Cement Containing Lactoferrin. Key Eng. Mater..

[29] Kim S.E., Lee D.-W., Yun Y.-P., Shim K.-S., Jeon D.I., Rhee J.-K., Park K. (2016). Heparin-immobilized hydroxyapatite nanoparticles as a lactoferrin delivery system for improving osteogenic differentiation of adipose-derived stem cells. Biomed. Mater..

[30] Dai R., Wang Z., Samanipour R., Koo K.-I., Kim K. (2016). Adipose-Derived Stem Cells for Tissue Engineering and Regenerative Medicine Applications. Stem Cells Int..

[31] Calabrese G., Giuffrida R., Forte S., Fabbi C., Figallo E., Salvatorelli L., Memeo L., Parenti R., Gulisano M., Gulino R. (2017). Human adipose-derived mesenchymal stem cells seeded into a collagen-hydroxyapatite scaffold promote bone augmentation after implantation in the mouse. Sci. Rep..

[32] Krishna K.A., Vishalakshi B. (2017). Gellan gum-based novel composite hydrogel: Evaluation as adsorbent for cationic dyes. J. Appl. Polym. Sci..

[33] Anghel L., Erhan R. (2018). Structural Aspects of Lactoferrin and Serum Transferrin Observed by FTIR Spectroscopy. Chem. J. Mold..

[34] Olimpio R.M.C., De Oliveira M., De Síbio M.T., Moretto F.C.F., Deprá I.C., Mathias L.S., Gonçalves B.M., Rodrigues B.M., Tilli H.P., Coscrato V.E. (2018). Cell viability assessed in a reproducible model of human osteoblasts derived from human adipose-derived stem cells. PLoS ONE.

[35] Chang H.-I., Wang Y., Eberli P.D. (2011). Cell Responses to Surface and Architecture of Tissue Engineering Scaffolds. Regenerative Medicine and Tissue Engineering—Cells and Biomaterials.

[36] Deville S., Saiz E., Tomsia A.P. (2006). Freeze casting of hydroxyapatite scaffolds for bone tissue engineering. Biomaterials.

[37] Bobbert F.S.L., Zadpoor A.A. (2017). Effects of bone substitute architecture and surface properties on cell response, angiogenesis, and structure of new bone. J. Mater. Chem. B.

[38] Ryan A.J., Gleeson J.P., Matsiko A., Thompson E.M., O’Brien F.J. (2015). Effect of different hydroxyapatite incorporation methods on the structural and biological properties of porous collagen scaffolds for bone repair. J. Anat..

[39] Dey S., Pal S. (2009). Evaluation of Collagen-hydroxyapatite Scaffold for Bone Tissue Engineering. IFMBE Proceedings, Proceedings of the 13th International Conference on Biomedical Engineering, Singapore, 3–6 December 2008.

[40] Correlo V.M., Pinho E.D., Pashkuleva I., Bhattacharya M., Neves N.M., Reis R.L. (2007). Water Absorption and Degradation Characteristics of Chitosan-Based Polyesters and Hydroxyapatite Composites. Macromol. Biosci..

[41] Montesi M., Panseri S., Iafisco M., Adamiano A., Tampieri A. (2015). Effect of hydroxyapatite nanocrystals functionalized with lactoferrin in osteogenic differentiation of mesenchymal stem cells. J. Biomed. Mater. Res. Part A.

[42] Cabañas M.V., Peña J., Román J., Ramírez-Santillán C., Matesanz M.C., Feito M.J., Portolés M.T., Vallet-Regí M. (2014). Design of tunable protein-releasing nanoapatite/hydrogel scaffolds for hard tissue engineering. Mater. Chem. Phys..

